# Ethical Applications of Artificial Intelligence: Evidence From Health Research on Veterans

**DOI:** 10.2196/28921

**Published:** 2021-06-02

**Authors:** Christos Makridis, Seth Hurley, Mary Klote, Gil Alterovitz

**Affiliations:** 1 National Artificial Intelligence Institute Department of Veterans Affairs Washington, DC United States; 2 Stanford Digital Economy Lab Stanford University Stanford, CA United States; 3 WP Carey School of Business Arizona State University Tempe, AZ United States; 4 Office of Research & Development Department of Veterans Affairs Washington, DC United States; 5 Boston Children’s Hospital Harvard Medical School Boston, MA United States

**Keywords:** artificial intelligence, ethics, veterans, health data, technology, Veterans Affairs, health technology, data

## Abstract

**Background:**

Despite widespread agreement that artificial intelligence (AI) offers significant benefits for individuals and society at large, there are also serious challenges to overcome with respect to its governance. Recent policymaking has focused on establishing principles for the trustworthy use of AI. Adhering to these principles is especially important for ensuring that the development and application of AI raises economic and social welfare, including among vulnerable groups and veterans.

**Objective:**

We explore the newly developed principles around trustworthy AI and how they can be readily applied at scale to vulnerable groups that are potentially less likely to benefit from technological advances.

**Methods:**

Using the US Department of Veterans Affairs as a case study, we explore the principles of trustworthy AI that are of particular interest for vulnerable groups and veterans.

**Results:**

We focus on three principles: (1) designing, developing, acquiring, and using AI so that the benefits of its use significantly outweigh the risks and the risks are assessed and managed; (2) ensuring that the application of AI occurs in well-defined domains and is accurate, effective, and fit for the intended purposes; and (3) ensuring that the operations and outcomes of AI applications are sufficiently interpretable and understandable by all subject matter experts, users, and others.

**Conclusions:**

These principles and applications apply more generally to vulnerable groups, and adherence to them can allow the VA and other organizations to continue modernizing their technology governance, leveraging the gains of AI while simultaneously managing its risks.

## Ethical Applications of Artificial Intelligence in Veterans’ Health Research

There is increasing recognition that artificial intelligence (AI) offers significant potential to help or harm the world. Much like other technologies, ranging from the internet to computers, AI is neither bad nor good: the impact of AI depends on how its users wield it. Already, there is an emerging body of AI use cases in health care [1,2], including for vulnerable groups and veterans [3], that are increasingly originating from populations the federal government considers to be “potentially vulnerable patient populations” [4]. These groups can be especially sensitive to adoption of technology; therefore, additional scrutiny is required around the ethical underpinnings and likely causal effects on these groups. In this sense, the question is not whether the federal government should engage AI for broader social benefit, but how it can do so using a values-based framework to guide AI applications and their continued research and development.

At least since the publication of the Belmont Report [5], there has been general recognition in the federal government of three principles that guide the introduction of new technologies to this day. First, *respect for persons* details that individual autonomy and privacy must be protected. Second, *beneficence* states that technologies should be designed to maximize the potential net benefits to society, safeguarding against potential harms and long-term consequences. Third, *justice* ensures that there are equitable benefits from research. That is, when individual data is collected, it must be used to benefit those individuals. Although the Belmont Report focused on biomedical technologies, they exhibit many similarities with AI, particularly in terms of their ethical implications and long-lasting impacts.

The primary contribution of this commentary is to explore the ethical applications of AI by building on the Belmont Report and relating it with the principles established in the recent executive order on trustworthy AI. Although there has been a recognition of data ethics and privacy within the US federal government, a new challenge has emerged: how can the federal government balance between the competing priorities of stewarding sensitive data and using AI to analyze it to drive veteran outcomes?

To answer this question, we apply the perspective of the Veterans Health Administration, within the Department of Veterans Affairs (VA), which has the largest integrated health care system in the United States and has pioneered several technological aspects now widely seen in this country, such as electronic health records (EHRs). Additionally, more than half of physicians training within the United States receive some training at a VA medical center. By evaluating uses for AI and implications in health care, veteran input and priorities can be proactively developed to enhance care. For example, the VA is already using AI to facilitate early detection of cancer [3], detection of acute kidney injury [6], and prediction of loneliness and declines in mental health [7,8]. These examples all highlight the ways that AI can be used to advance patient outcomes; however, they also point toward data privacy and trust considerations.

The recent executive order, “Promoting the Use of Trustworthy Artificial Intelligence in Government” [9], provides a framework for the VA to move forward with using AI to improve veteran health on a larger and more systematic scale. We focus on three principles that are especially relevant to the advancement of the health and well-being of veterans: (1) purposeful and performance-driven; (2) accurate, reliable, and effective; and (3) understandable.

## 1. Purposeful and Performance Driven

…seek opportunities for designing, developing, acquiring, and using AI, where the benefits of use significantly outweigh the risks and the risks are assessed and managed

9

The VA is working to employ AI in high priority areas where there is robust opportunity to advance veteran health outcomes. Recent work indicates that difficulty in transitioning to civilian life is a critical factor underlying negative mental health outcomes in veterans. For example, Makridis and Hirsch [10] have documented a deterioration in labor market outcomes among veterans over the past decade, showing that veterans are increasingly concentrated in metropolitan areas with lower wage and employment growth. Moreover, Makridis et al [11] show that socioeconomic factors are the largest predictors of mental health outcomes among veterans, dwarfing the contribution of location and demographic-specific features. Intuitively, because a significant amount of time is allocated toward work activities, the absence of purpose and self-efficacy in the workplace, especially after coming from a mission-driven environment in the armed services, will impact veterans’ mental health.

AI can be part of the solution. To the extent that veteran records from combat are combined with self-assessments of skills and career preferences, and these data could be comprehensively gathered and harmonized, researchers could use methods from AI to provide veterans with personalized recommendations regarding not only potential job fits but also counseling over the course of their careers. One of the sources of low engagement among employees is a feeling of plateauing and helplessness; therefore, AI-driven recommendations regarding how to optimize career mobility and human capital development would provide veterans with actionable steps to continuously acquire and apply new skills at work.

Another prime example involves personalizing feedback to veterans about how to live healthier lives. End-of-life care is one of the largest sources of health care expenditures. For example, Riley and Lubitz [12] estimate that a quarter of all Medicare spending goes toward care for people during their last year of life. These resources could be more impactful if they were allocated more toward preventative care earlier in life. Using deep learning methods, Ahadi et al [13] illustrate how biological data can be used for longitudinal profiling. Implementing this algorithm, combined with EHRs at the VA, offers the potential to provide practical advice about how to live more productive and happier lives, raising both economic and social well-being.

Veterans in rural areas face challenges accessing care due to a paucity of rural treatment facilities. AI, implemented along with smart devices (eg, smart wearables), could allow for remote monitoring of rural veterans’ health and enable smart devices to alert veterans of health concerns. Recent evidence indicates that AI may be able to predict a person’s mental state, including the likelihood of suicide, raising the likelihood that smart devices could be used for predicting and intervening in veteran suicide [7,8].

However, the benefits of AI depend on ethical implementation. Risks associated with AI implementation need to be thoroughly assessed and managed. If, for example, privacy is disrespected, public trust and confidence, particularly among those who have already sacrificed so much for their country, would be undermined. This is extremely important at the VA, where sensitive data, which is under continuous reassessment and review, is routinely collected from veterans. Moreover, researchers must be cognizant of the potential for replicating sources of bias when training their AI algorithms. That is, researchers must investigate the data and model to, at least qualitatively, assess whether there are potential biases that could lead to error replication through the AI-driven recommendations. For example, one possibility is that samples are not representative of the entire population of veterans [14], particularly those who do not feel comfortable using technology. Researchers must also ensure that AI-driven insights are derived from representative samples that reflect the diversity of experiences, attitudes, ethnic, and gender composition among veterans. Recent evidence, for example, highlights the lack of diversity in many health care databases as a major limitation [15].

## 2. Accurate, Reliable, and Effective

…ensure that their application of AI occurs in well-defined domains, and is accurate, reliable, effective, and fit for intended purposes

9

The VA is well-equipped to ensure the accuracy, reliability, and effectiveness of AI applications in health and well-being. The VA has collected and catalogued over two petabytes of data, including data on veteran health, prescription data, and inpatient and outpatient services, among others. Further, the VA established the Million Veteran Program, which characterizes, through a consented cohort of subjects, the confluence of genes, lifestyle, and military exposure on veteran health outcomes. This breadth of data paves the way for algorithms that promote personalized medicine based upon life experience and genetic factors. In particular, the plethora of data at the VA can be leveraged to train high-quality algorithms to serve veteran needs.

Concerns have been raised over whether AI algorithms will be effective and generalize beyond the training set originally used to develop machine learning (ML) algorithms [14]. Importantly, the VA’s data sets are generated from VA centers across the country and, in principle, data should accurately capture the diverse spectrum of veterans. Therefore, AI algorithms trained on these data should prove to be reliable even when implemented in varied VA centers throughout the United States. However, cautious implementation and monitoring is necessary to ensure that each developed AI algorithm is beneficial at VA centers.

Although the VA database spans millions of veterans, there are still many veterans who are not included in the system. For example, homelessness is a large challenge for the veteran population, and if these veterans are not included within the VA system, they cannot receive the available benefits and treatment [16]. Our internal calculations from the American Community Survey conducted by the Census Bureau suggest that there are roughly 18 million veterans in the United States, whereas the VA only covers roughly 9 million of them [17]. To ensure that AI applications produce reliable recommendations for all veterans, it is important to ensure that the data being fed into predictive models is representative.

In addition to the importance of maintaining a representative sample, researchers and clinicians must use appropriate AI techniques. One particularly large challenge with clinical decision support tools and the use of electronic health records is the presence of missing data and small sample sizes. While sample size is less of a challenge within the VA because of the size of its EHR database, missing data can be a source of bias if they are not missing at random [18]. Some ML techniques, such as gradient boosting and decision trees, can deal well with missing data; however, researchers need to be careful about applying ML and automation in these environments. There is also a well-known bias that can emerge against specific groups, whether by race or even socioeconomic status, which can be propagated at scale if ML algorithmics are not trained and “de-biased” properly [19]. However, it is becoming clear that researchers developing predictive models for clinical use need to transcend traditional conversations about algorithmic bias and think harder about the broader and structural forces that are at play in the observed phenomena [20].

## 3. Understandable

…ensure the operations and outcomes of their AI applications are sufficiently interpretable and understandable by subject matter experts, users, and others as appropriate

9

A concern for AI development is the necessity for algorithms to be explainable. Explainability is the concept that users should be able to understand how algorithms function, and it is conceptualized along a continuum where relatively simple algorithms based upon branching decision trees and linear regression are feasible to understand [21]. However, the use of deep neural networks (DNNs), where decision-making is spread across multiple layers of interconnected decision-making nodes, currently produces results that are difficult to accurately interpret. Although DNNs provide great utility in analyzing complex data sets, there is concern over the “black box” nature of DNNs, although new methods are being developed to provide explainability to DNNs [22]. Explainable algorithms will foster trust in AI by both clinicians and patients at the VA.

These new principles for the promotion of trustworthy AI build upon an existing framework developed in the VA, Ethical Principles for Access to and Use of Veteran Data [23], that safeguards veterans and their data and ensures that veterans benefit from research. In other words, research is not an end in and of itself—it is a means toward delivering value to veterans. Moreover, these principles are rooted in the legacy of the Belmont Report from 1979 [5], which emphasized privacy, beneficence, and justice in applications of technology. At its root, technology exists to help improve well-being, whether through heightened productivity or quality of the services provided. Together, these principles provide a signpost for clinicians and researchers to work collaboratively so that AI is developed and deployed for social good, especially for vulnerable groups and veterans.

Moreover, these ethical principles developed and operationalized within the VA can be extended across the broader health care sector. For example, large university hospital systems that exist within the research ecosystem can adopt these ethical principles to guide their strategic investments and the development and deployment of AI tools. In fact, these university ecosystems have many similarities to the VA because they bring together a combination of researchers and clinicians under a common umbrella and institutional resources. Researchers and clinicians can work hand-in-hand to ensure that research and development investments are fundamentally driven by areas of great need and potential impact.

These processes for the development and application of ethical AI extend beyond veterans. In particular, members of any vulnerable group are beneficiaries of adherence to these processes because, by definition, they may find it harder to benefit from AI. For example, while AI is also leading to the invention of new jobs and tasks in the labor market, AI also reduces the demand for other skills that are more routine and manual, which may affect veterans more if they are concentrated in those types of jobs and occupations. In this sense, applications of AI aimed at improving the transition of service members into the civilian sector could not only help veterans directly by, for example, providing them with tools to more efficiently match into jobs that suit their preferences and abilities, but could also improve trust and confidence in the benefits of AI. Moreover, other vulnerable groups likely face similar challenges; therefore, processes for the development and application of AI would help them too.

Our paper explains some of the most important ingredients for ensuring that AI advances are applied in ways that promote improved veteran outcomes. Furthermore, the VA could serve as a model organization, protecting VA patient data and leveraging it for their good and ultimately cutting health care costs, increasing efficiency, and enhancing health care for veterans. If the United States can successfully scale AI under a technology governance structure using these principles, the possibilities are limitless.

## Abbreviations

AI: artificial intelligence
DNN: deep neural network
EHR: electronic health record
ML: machine learning
VA: Department of Veterans Affairs
